# Radiotherapy and "new" drugs-new side effects?

**DOI:** 10.1186/1748-717X-6-177

**Published:** 2011-12-21

**Authors:** Maximilian Niyazi, Cornelius Maihoefer, Mechthild Krause, Claus Rödel, Wilfried Budach, Claus Belka

**Affiliations:** 1 Department of Radiation Oncology, Ludwig-Maximilians-University Munich, Marchioninistr. 15, 81377 München, Germany; 2 Klinik und Poliklinik für Strahlentherapie und Radioonkologie, Universitätsklinikum Carl Gustav Carus, Technische Universität Dresden, Fetscherstraße 74, 01307 Dresden, Germany; 3 Klinik für Strahlentherapie und Onkologie, Johann Wolfgang Goethe Universität Frankfurt, Theodor-Stern-Kai 7, 60590 Frankfurt am Main, Germany; 4 Klinik und Poliklinik für Strahlentherapie und Radioonkologie, Heinrich Heine Universität Düsseldorf, Moorenstr. 5, 40225 Düsseldorf, Germany

**Keywords:** radiotherapy, molecular targeted drugs, antibodies, TKI, toxicity

## Abstract

**Background and purpose:**

Targeted drugs have augmented the cancer treatment armamentarium. Based on the molecular specificity, it was initially believed that these drugs had significantly less side effects. However, currently it is accepted that all of these agents have their specific side effects. Based on the given multimodal approach, special emphasis has to be placed on putative interactions of conventional cytostatic drugs, targeted agents and other modalities. The interaction of targeted drugs with radiation harbours special risks, since the awareness for interactions and even synergistic toxicities is lacking. At present, only limited is data available regarding combinations of targeted drugs and radiotherapy. This review gives an overview on the current knowledge on such combined treatments.

**Materials and methods:**

Using the following MESH headings and combinations of these terms pubmed database was searched: Radiotherapy AND cetuximab/trastuzumab/panitumumab/nimotuzumab, bevacizumab, sunitinib/sorafenib/lapatinib/gefitinib/erlotinib/sirolimus, thalidomide/lenalidomide as well as erythropoietin. For citation crosscheck the ISI web of science database was used employing the same search terms.

**Results:**

Several classes of targeted substances may be distinguished: Small molecules including kinase inhibitors and specific inhibitors, antibodies, and anti-angiogenic agents. Combination of these agents with radiotherapy may lead to specific toxicities or negatively influence the efficacy of RT. Though there is only little information on the interaction of molecular targeted radiation and radiotherapy in clinical settings, several critical incidents are reported.

**Conclusions:**

The addition of molecular targeted drugs to conventional radiotherapy outside of approved regimens or clinical trials warrants a careful consideration especially when used in conjunction in hypo-fractionated regimens. Clinical trials are urgently needed in order to address the open question in regard to efficacy, early and late toxicity.

## Background and purpose

Several new anti-cancer drugs have recently entered clinical practice in oncology. Among those, especially targeted drugs are promising therapeutic candidates with a comparatively low toxicity profile. At present, these drugs are often applied in palliative treatment situations for metastasized diseases. In addition, targeted agents are a substantial part of many multimodal oncologic treatment schedules. Thus the risk of parallel use of both radiotherapy and targeted drug is given. With few exceptions, the toxicity of any combination of targeted drugs with radiotherapy has not yet been studied in detail.

Key cellular signalling pathways [1] are responsible for the response of normal tissue and tumour cells to radiation therapy [2]. Although some of the anti-cancer targets are specific for neoplastic signalling, there is considerable overlap between neoplastic signalling and normal cellular signalling. In this regard, several putative interactions with radiation triggered signalling in normal issues exist and thus [3,4] influences of targeted drugs on normal tissue reactions cannot be excluded [5-7].

The present article reviews the existing data on the toxicity profile and efficacy (if available) of targeted drugs when applied concurrently to radiotherapy.

## Methods and materials

Using the following MESH headings and combinations of these terms, pubmed database was searched for randomized, prospective and retrospective trials as well as case reports (all sample sizes were considered):

1. Radiotherapy AND cetuximab/trastuzumab/panitumumab/nimotuzumab

2. Radiotherapy AND bevacizumab

3. Radiotherapy AND sunitinib/sorafenib/lapatinib/gefitinib/erlotinib/sirolimus

4. Radiotherapy AND thalidomide/lenalidomide.

5. Radiotherapy AND erythropoietin

For citation crosscheck, the ISI web of science database was used employing the same search terms. A focus was put on prospective or phase I/II trials; if available, some smaller case studies or case reports were included if higher toxicities were reported.

In general, grade III + IV toxicities are reported. For cetuximab, focus was set on larger phase III trials and those reporting trials specifically reporting toxicities. In addition, key reviews focusing on the use of targeted drug in oncology were screened in order to identify clinically relevant drugs [8].

## Results

### Antibodies

#### Cetuximab

Cetuximab is a monoclonal chimeric antibody directed against the epidermal growth-factor receptor (EGF-R). It has first been approved for treatment of locally advanced or metastatic colorectal cancer (k-ras wildtype) refractory to irinotecan [9]. Regarding radiotherapy, it has been approved for head-and-neck cancer as an alternative to concomitant chemotherapy [10]; in the given phase III trial overall survival of patients who were treated by radiotherapy and cetuximab was improved compared to patients who underwent radiotherapy alone. Cetuximab also has a proven efficacy in locally advanced or metastatic head-and-neck cancer in combination with 5-FU/cisplatin [11].

Thus several pre-clinical and clinical studies have provided evidence for the efficacy of cetuximab in combination with radiotherapy [12-17]. Nevertheless, several reports are available pointing to increased skin toxicity after combining cetuximab with radiotherapy [18-27] (a complete overview is given in Table 1). The initial publication on the combined use by Bonner and colleagues reported an increased incidence of an acneiform rash [10]. However, in single cases more severe complications occurred [19]. A recent retrospective matched-pair evaluation of acute toxicity during cis-platinum-based radio-chemotherapy versus radiotherapy with simultaneous cetuximab treatment showed significantly higher grade 3 oral mucositis and dermatitis as well as a higher risk of weight loss (> 10%) and of enteral feeding requirement in the cetuximab-group. However, this may be outweighed by the higher risk of haematological toxicity by radio-chemotherapy. In keeping with this, higher compliance rate with less treatment interruptions in the cetuximab-treated group was described [26]. In trials on thoracic [28,29] or pelvic radiotherapy with cetuximab increased rates of skin toxicity were not observed.

**Table 1:** Studies on monoclonal EGFR antibodies

Substance	Author(s)	Year	Study type	N	Tumour	RT dose/ChTx/technique	Toxicity
Cetuximab	Bonner et al. [10]	2006	Phase III	211 (cetux-arm)	LA-HNSCC	70-78.8 Gy (hyper-fractionated)	Significant differences or trend in arms: 8% grade III-V acneiform rash, 1% grade III-V voice alteration, 1% grade III-V infusion reaction
	Koutcher et al. [24]	2009	Retrospective	115	LA-HNSCC	66 Gy/69.96 Gy	3% grade IV radio-dermatitis, 19% grade III radio-dermatitis
	Studer et al. [25]	2011	Prospective	99	HNSCC	66-70 Gy, 30/99 switch from Cis	34% grade II/IV dermatitis
	Hallqvist et al. [28]	2010	Phase II	75	NSCLC	68 Gy, Ind. ChTx Doc/Cis + concomitant Cetux	1% grade V pneumonitis, 4% grade III pneumonitis, 5% grade III + IV hypersensitivity, 15% grade III + IV febrile neutropenia, 4% III skin reactions
	Jensen et al. [122]	2010	Retrospective	73	HNSCC	22 pts Re-RT (50-60 Gy), 66-70 Gy	5% grade III allergic reaction, 4% grade III acneiform rash
	Garcia-Huttenlocher et al. [123]	2009	Retrospective	65	HNSCC	Median 66 Gy (IMRT)	Grade III: skin toxicity 28%, mucositis 25%
	Rödel et al. [115]	2008	Phase I/II	12/48	Rectal cancer	50,4 Gy +Capecitabine + Oxaliplatin	Phase II only Grade IV/V: Leukopenia, thrombocytopenia, Diarrhea, Creatinine elevation, e-lyte derivation, infection each 2%
	Safran et al. [31]	2008	Phase II	60	Esophago-gastric-cancer	Cetux/Carbo/Tax + 50.4 Gy	23% grade III rash, 15% grade III/IV esophagitis, 5% III + IV hypersensitivity, 3% grade IV neutropenia (10% grade III), 2% IV anemia (8% grade III)
	Jatoi et al. [124]	2010	Phase II	57	NSCLC	60 Gy	2% grade IV each: dysphagia/hypomagnesemia/dyspnea/headache/thrombosis/GI hemorrhage, 7% grade III rash
	Horisberger et al. [30]	2009	Phase II	50	Rectal cancer	50,4 Gy + Capecitabine + Irinotecan	Leukopenia 4% grade III and IV each Grade III: Diarrhea 60%, abdominal pain 8%, ALAT/ASAT elevation 20%, Acneiform skin rash 12%, anemia, nausea/vomiting, bilirubin elevation, proctitis each 4%
	Koutcher et al. [125]	2011	Retrospective	49	LA-HNSCC	69.96 Gy (IMRT) (comparison vs. concomitant Cis)	20% late grade III + IV toxicity
	Walsh et al. [26]	2011	Retrospective	48 (14 excluded because of SIB)	HNSCC	Cis vs. Cetux (66-70 Gy)	44% ≥ grade III skin toxicity, 52% ≥ grade III mucositis, 6% ≥ grade III acneiform rash
	Buiret et al. [126]	2010	Retrospective multicenter	46	HNSCC	Ind. ChTx Doc/Cis/5-FU, RIT (70 Gy)	No grade IV toxicity
	Garcia-Huttenlocher et al. [127]	2008	Retrospective	46	HNSCC	Median 66 Gy (IMRT)	20% grade III skin toxicity, 4% grade III mucositis
	Merlano et al. [128]	2010	Phase II	45	HNSCC	Up to 70 Gy, three cycles Cis/5-FU, split course RT, RT + cetux	2% grade IV leukopenia (38% grade III), 7% grade IV neutropenia (33% grade III), 2% grade IV thrombopenia (13% grade III), 36% grade IV stomatitis (29% grade III), 73% grade III radiodermatitis, 7% grade III rash
	Koukourakis et al. [60]	2010	Phase I	43	LA-HNSCC	21 × 2.7 Gy (56.7 Gy) + amifostine + Cis	16% grade III + IV mucositis, 2% grade III + IV skin toxicity
	Suntharalingam et al. [129]	2011	Phase II	43	LA-HNSCC	70,2 Gy (3D/IMRT) + Paclitaxel, Carboplatin,	Grade 3 mucositis (79%), rash (9%), leukopenia (19%), neutropenia (19%), and RT dermatitis (16%)
	De Vita et al. [130]	2011	Phase II	41	Esophageal cancer	Ind. FOLFOX4 + 50.4 Gy/Cetux	30% grade II/IV skin toxicity/neutropenia
	Bertolini et al. [117]	2009	Phase II	40	LA rectal cancer	50-50.4 Gy + neoadj. Cetux/Cetux + 5-FU concomitant	8% grade III/IV skin rash, 8% grade III/IV hypersensitivity, 13% grade III/IV GI toxicity, 3% grade III/IV febrile neutropenia
	Kim et al. [98]	2011	Phase II	40	Rectal cancer	Capecitabine + Cetux + Irinotecan + 50.4 Gy	3% grade IV leukopenia, 3% grade III rash
	Machiels et al. [119]	2007	Phase I/II	40	Rectal cancer	45 Gy + Capecitabine	3% grade III/IV allergic reaction, 3% grade III/IV dermatitis
	Argiris et al. [131]	2010	Prospective	39	LA-HNC	Induction Docetaxel/Cis/Cetux + concurrent Cisplatin/Cetux/70-74 Gy-RT	Grade III/IV: oral mucositis 46%, Anemia 21%, in-field dermatitis 23%, Dysphagia 41%, Thrombocytopenia 10%, Neutropenia 31%, febrile neutropenia 5%, infection 18%, fatigue 13%, nausea 10%, vomiting 3%, renal failure 3%, DVT 5%, bleeding 5%.
	Velenik et al. [118]	2010	Phase II	37	Rectal cancer	45 Gy RT + capecitabine (neoadjuvant)	Grade III: diarrhea 11%, anorexia 3%, hepatotoxicity 3%, in-field-dermatitis 16%, infection 3%, hypersensitivity 5%.
	Heron et al. [132]	2011	Matched pair retrospective	35	HNSCC	SBRT Re-RT	No significant increase grade III + IV
	Birnbaum et al. [133]	2010	Phase I	32	LA-HNSCC	66-72 Gy, Ind. Cetux + Carbo/Tax/Cetux concomitant	3% grade III allergic reaction, 3% grade IV metabolic symptom, 69% grade III + IV mucositis, 3% grade IV dysphagia, 59% pts grade III + IV skin toxicity
	Jensen et al. [134]	2011	Phase II	30	NSCLC	66 Gy (IMRT)	Pulmonary embolism 3% grade III + 3% grade V endocarditis and myocardial infarction grade V each 3%, 13% grade III/IV pneumonia esophagitis, diarrhea, DVT, exacerbation of COPD, urosepsis, pericardial effusion, pneumonitis grade III each 3%
	Ruhstaller et al. [135]	2011	Phase IB/II	28	Esophageal cancer	Induction ChTx Cis/docetaxel + neoadjuvant RCh-immunotherapy	25% grade III/IV esophagitis, 4% grade III/IV rash
	Pfister et al. [136]	2006	Phase I	22	LA-HNSCC	70 Gy RT + Cisplatin	Study closed due to significant AEs. Grade V pneumonia and one death of unknown cause, Grade IV: MI 5%, arrhythmia 5%, metabolic 5%, infection 5%
	Hofheinz et al. [137]	2006	Phase I	20	Rectal cancer	Capecitabine + Irinotecan + 50.4 Gy	No grade IV, no rash, 20% grade III diarrhea
	Kuhnt et al. [138]	2010	Phase I	18 (16 eligible)	LA-HNSCC	HART (70.6 Gy) + Cis	56% grade III mucositis, 38% ≥ grade III radiodermatitis, 25% ≥ grade III neutropenia, 6% grade III rash
	Pryor et al. [22]	2009	Prospective	13	HNSCC	70 Gy	46% ≥ grade III acneiform rash, 77% ≥ grade III dermatitis
	Hughes et al. [29]	2008	Phase I	12	NSCLC	64 Gy	Grade III fatigue, pneumonitis each 8% Grade V Infection 8%
	Zwicker et al. [139]	2011	Phase II	10	HNSCC	IMRT 50.4 Gy Re-RT + Cetux	10% grade V mucositis, 10% grade III mucositis, 10% grade IV erythema (20% grade III), 20% grade III acneiform rash
	Jensen et al. [140]	2010	prospective	9	Adenoid cystic carcinoma of HN	5/9: re-RT: median 50,4 Gy, median 65 Gy otherwise (IMRT or C-12 boost)	Grade III Mucositis and Grade III Dysphagia
	Balermpas et al. [141]	2009	Prospective	7	HNSCC	Re-irradiation 50,4 Gy-54,0 Gy	New acute side effects: Grade III: pain 14%, mucositis 71%. Dysphagia 57%. Xerostomia 14%. Fibrosis 14%, acneiform rash 29%.
	Berger et al. [19]	2008	Case report	1	HNSCC	72 Gy., regimen change to cetuximab from 5-FU/MMC	Grade IV Dermatitis
Trastuzumab	Halyard et al. [48]	2009	Phase III	1503	Breast cancer	Median 50,4 Gy, previous OP + ChTx	Skin toxicity grade III: 4% (simultaneous) -6% (adjuvant), cardiac events 2% (simultaneous)-3% (adjuvant)
	Belkacemi et al. [49]	2008	Multicentric study	146	Breast cancer	Median 50 Gy	> grade II esophagitis (12%), 1 pt grade III esophagitis, 5% grade III dermatitis, ≥ grade II LVEF (10%)
	Caussa et al. [142]	2011	Prospective	106	Breast cancer	50 Gy (2 Gy) + 16 Gy boost	2% grade III skin reaction, 1% grade III esophagitis
	Anderson [143]	2009	Matched case control study	85	Breast cancer	n. r.	Grade III dermatotoxicity 2%, 1% ≥ grade II LVEF decrease (reversible)
	Shaffer et al. [50]	2009	retrospective	44	Breast cancer	40-50.4 Gy	In 14% stopped because of cardiac toxicity
	Chargari et al. [144]	2011	Phase I	31	Brain mets breast cancer	30 Gy (3 Gy) WBRT	No grade > II
	Horton et al. [145]	2010	Phase II	12	Locally recurrent breast cancer	50 Gy, ChTx refractory	17% grade III skin toxicity, 8% grade III lymphopenia, no cardiac toxicity
Panitumumab	Pinto et al. [35]	2011	Phase II	60	Rectal cancer	5-fluorouracil-oxaliplatin + RT	Grade 3-4 toxicity: diarrhea (39%, one toxic death), cutaneous reactions (19%), nausea, neutropenia (2%), others.
	Wirth et al. [34]	2010	Phase I	19	LA-HNSCC	70 Gy + Carbo/Tax (2 Gy) IMRT	1 pt grade III febrile neutropenia, 84% grade III + IV mucositis, 95% grade III dysphagia, 42% grade III dermatitis, 11% grade III rash, 21% grade III nausea
Nimotuzumab (h-R3)	Rodriguez et al. [38]	2010	Prospective randomized	106	HNSCC	n. r.	III/IV not reported
	Crombet et al. [146]	2004	Phase I	24	HNSCC	66 Gy (2 Gy)	4% grade III somnolence, 13% grade III dysphagia, 21% grade III mucositis, 13% grade III dermatitis, 4% grade III laryngitis
	Bebb et al. [39]	2011	Phase I	18	NSCLC	36/30 Gy (3 Gy)	50% grade III + IV
	Choi et al. [40]	2010	Phase I	15	NSCLC	36/30 Gy (3 Gy)	7% grade IV febrile neutropenia/pneumonia, 40% grade III lymphopenia

N-number of patients, pt(s)-patient(s), n. r.-not reported, ChTx-chemotherapy, HCC-hepatocellular carcinoma, RCC-renal cell cancer, GBM-glioblastoma multiforme, DVT-deep vein thrombosis, Fx-fractions, SRS-stereotactic radiosurgery, DLT-dose limiting toxicity, LA-locally advanced, Gem-gemcitabine, Tax-Taxol (paclitaxel), Tx-therapy, TMZ-temozolomide, PCP-Pneumocystis pneumonia, Cis-cisplatin, Eto-etoposide, Doc-docetaxel

No other risks regarding additional or increased side effects concerning connective tissue, CNS [30-32] or peripheral nerves have been described so far in small early-phase clinical trials.

#### Panitumumab

Similar to cetuximab, panitumumab is a monoclonal antibody directed against EGF-R with a putatively higher affinity and less toxicity due to its non-chimeric design. It has been approved for stage IV colorectal cancer refractory to FOLFOX or FOLFIRI [33].

Only data from a single phase I study [34] and a single phase II trial described effects of a combination of panitumumab with a 5-FU/oxaliplatin-containing radio-chemotherapy for rectal cancer [35]. Pre-clinical data suggest a comparable efficacy to cetuximab [36]. Concerning toxicity, no additional toxicity was observed when combined with radiotherapy. The phase II trial reported one toxic death from diarrhea and a relatively high rate of grade III/IV diarrhea (39%) compared to the classical CAO/ARO/AIO-94 trial [37]. However, based on the design of the trial it is not possible to precisely attribute the side effects to any of the components of the given protocol.

#### Nimotuzumab

Nimotuzumab is another humanized therapeutic monoclonal antibody directed against EGF-R not yet been approved by the authorities in Europe. There are three small phase I trials testing radiotherapy and nimotuzumab in head-and-neck cancer as well as NSCLC patients; an increased rate of skin toxicity was observed [38-40]. The other larger phase II trial by Rodríguez and colleagues was prospectively randomized and 106 head-and-neck cancer patients were included [38]. No grade III or IV toxicity has been observed.

The data available suggest that the combination of cetuximab with radiation may lead to an increased rate of mucosal- and skin toxicity when applied together with radiation for the treatment of head-and-neck cancer. No such problems have been reported in other organ regions. It is unclear in how far this is an epitope-specific side effect-only limited data are available regarding similar effects after the combined use of panitumumab and nimotuzumab.

### Anti -Her2/neu antibody trastuzumab

Trastuzumab is a humanized monoclonal antibody directed against the epidermal growth-factor-receptor Her-2/neu. It is approved for the treatment of metastatic her-2/neu-positive breast cancer as well as for the adjuvant treatment of her-2/neu-positive breast cancer in combination with chemotherapy [41-44].

Cardiac toxicity is a rare, but well described adverse effect of trastuzumab-especially with or after the treatment with anthracyclins [45-47]. As cardiac toxicity is also of concern in thoracic radiotherapy, the question of an increased toxicity has been raised. The largest trial focusing on side effects of the combined use of radiotherapy and trastuzumab is the phase III NLCCTG trial N9831 for adjuvant trastuzumab and radiotherapy including 1503 patients [48]. The trial did not reveal any significant differences in toxicity regarding skin, pneumonitis or cardiac events. Also, a French multicentric study [49] including 146 patients did not observe an increased cardiac toxicity. Another study retrospectively investigated the combinational approach of trastuzumab and radiotherapy including the internal mammary lymph nodes [50]. Again, no increased cardiac toxicity has been observed.

Thus, at present there are no strong indicators for an increased cardiac toxicity. However, follow-up periods are only sufficient for an estimation of early cardiac toxicity caused by trastuzumab, but not for an in-depth assessment of late radiation-induced cardiac effects.

Altogether, the current data suggest that the use of trastuzumab in a close time frame with radiotherapy may be safe. However, the reported studies might still reveal an increased cardiac toxicity, as minor vascular changes might lead to an increased mortality in long-term follow-up [51].

### Bevacizumab

Bevacizumab is a humanized monoclonal antibody against the vascular endothelial growth-factor (VEGF). So far, bevacizumab has been approved for the treatment of metastatic colorectal carcinoma, in combination with standard chemotherapy (5-FU, irinotecan, oxaliplatin or capecitabine). Bevacizumab has been approved for the treatment of metastatic non-squamous-cell bronchial carcinoma, for the treatment of renal cell cancer and for the treatment of glioblastoma multiforme (US only). The FDA has withdrawn the approval for first line treatment of metastatic HER-2/neu-negative breast cancer-however, the drug still is approved in Europe.

The most common side effects of bevacizumab alone include impaired wound healing, hypertension, bleeding problems as well as an increased risk of thromboembolic events.

One of the first publications to describe an increased risk of combining bevacizumab with radiotherapy reported on patients with ischemic bowel complications after the administration of radiotherapy followed by bevacizumab [52].

A phase II study combining neoadjuvant bevacizumab, capecitabine and radiotherapy for locally advanced rectal cancer revealed an increased rate of wound complications such as delayed healing and wound dehiscence [53]. The data are in line with a number of similar reports and case studies, supporting the interpretation that the combined use of bevacizumab with neoadjuvant radiotherapy is associated with an increased risk of postoperative complications [54-57]. However, this interpretation is not homogenously supported by all available data [58-61]. In terms of tumour response, the rate of pathological complete responses seems to be enhanced [53].

The use of bevacizumab, capecitabine and radiotherapy in patients with locally advanced pancreatic cancer was associated with an increased rate GI-bleeding and ulcerations (12%) [62]. These complications preferentially occurred in patients with a mucosal infiltration of the tumour. In a consecutive study -after excluding patients with mucosa infiltration-no such side effects were reported [63]. A similar study reported the combination of radiotherapy with bevacizumab-partly in a neoadjuvant setting- as "feasible" [64].

The combination of radiotherapy with simultaneous administration of bevacizumab was also tested for lung cancer [65,66]. In this setting, the occurrence of severe fistula leading to a discontinuation of both trials has been described [66].

In case of breast cancer the parallel combination of radiotherapy and bevacizumab had no significant side-effects in regard to lung and skin toxicity [65].

The treatment of malignant tumours of the brain has been subject to a variety of studies combining radiotherapy with bevacizumab with or without temozolomide; regarding progression-free survival, these trials suggest a benefit of the combined use [67]. No intra-cerebral bleeding has been reported, however cases of wound dehiscence of the previous operation have been documented [68-70]. A collection of case reports points towards increased late toxicity such as optic neuropathy and a single case of Brown-Séquard syndrome after a combination of bevacizumab with radiotherapy [71]. (a complete overview is given in Table 2).

**Table 2:** Studies on VEGF antibodies.

Substance	Author(s)	Year	Study type	N	tumour	RT dose/ChTx/technique	Toxicity
Bevacizumab	Vredenburgh et al. [147]	2010		125	Glioblastoma	59,4 Gy/Temozolomide	Grade III Thromboembolic events: 2%
	Crane et al. [63]	2009	Phase II	82	Pancreatic Cancer	50,4 Gy/Capecitabine	Toxicities possibly attributable to bevacizumab: GI bleeding: 6%. (d83,127,179, 180, 316); grade III, IV, V GI perforation: 4%. (d195,231,286) DVT grade III + IV: 4%; grade III hypertension: 2%;
	Lai et al. [72]	2010	Phase II	70	Glioblastoma	60 Gy/Temozolomide	Grade IV cerebrovascular ischemia: 9%; Grade III+IV CNS hemorrhage: 3%; Grade III+IV GI bleeding/perforation 6%; grade IIII Optic neuropathy: 1%. Grade III+IV venous Thrombosis/PE: 19%.
	Crane et al. [62]	2006	Phase I	48	Pancreatic Cancer	50,4 Gy/Capecitabine	Toxicities possibly attributable to bevacizumab: grade III+V ulceration with bleeding in RT field: 8%. (retrospectively fistulous connection identified in 4%) Grade III GI perforation: 4%; bleeding outside field): 4%; grade III hypertension: 2%.
	Seiwert et al. [148]	2008	Phase I	43	Head & Neck	63-72 Gy/5-FU and Hydroxyurea	Grade V bleeding events: 5%; grade V infection/sepsis: 7%, 2% unknown cause of death; grade III+ Thromboembolic events: 5% DVT, 2% stroke (leading to fatal sepsis, see above); fistula (due to radionecrosis or residual tumour): 12%. Tissue necrosis 9%.
	Spigel et al. [149]	2009	Phase II	A 29 B 5	SCLC	61,2 Gy/Carboplatin/Irinotecan (A-limited stage B-locally advanced)	A Grade IV+V tracheoesophageal fistula: 7%. Grade V aerodigestive hemorrhage. B Grade III tracheoesophageal fistula: 40%. Both studies closed due to toxicity.
	Willet et al. [58]	2009	Phase II	32	Rectal Cancer	50,4 Gy//5-FU	Grade III toxicities: GI abscess 3%, Hypertension 9%, radiation dermatitis: 6%; wound separation 3%. No grade IV.
	Dipetrillo et al. [55]	2012	Phase I	26	Rectal Cancer	50,4 Gy/FOLFOX	Grade III + IV Diarrhea: 42%; Bleeding (g3): 4%; g3 neuropathy: 4%; Radiation dermatitis G3: 8%; postoperative wound complications: 35%-the study was discontinued due to this toxicity.
	Crane et al. [53]	2010	Phase II	25	Rectal Cancer	50,4 Gy/Capecitabine	grade III perianal desquamation: 4%; 12% major surgical complications such as anastomotic dehiscence (4%), wound dehiscence (8%)
	Gutin et al. [68]	2009	Phase I	25	Glioblastoma/Anaplastic Gliomas	30 Gy/5 × 6 Gy	Grade IV Gastrointestinal bleeding: 4%, bowel perforation: 4%, wound healing complication: 4%. Grade III CNS hemorrhage: 4%.
	Koukourakis et al. [59]	2009	Phase I/II	22	Rectal Cancer	15 × 3,4 Gy/amifostine, capecitabine	Fistula: 9%, grade IV skin necrosis 5%.
	Niyazi et al. [69]	2010	Retrospective	20	Recurrent Glioblastoma	36 Gy	Grade IV wound healing complication: 5%. grade III DVT: 5%.
	Koukourakis et al. [150]	2011	Phase II	19	Rectal cancer	10 × 3, 4 Gy Amifostine/Capecitabine	Grade III diarrhea: 11%.
	Goyal et al. [65]	2010	Retrospective	14	Breast cancer	50 Gy + 10 Gy	No Grade III/IV toxicity (only acute toxicity assessed)
	Czito et al. [61]	2007	Phase I	11	Rectal Cancer	50,4 Gy/Oxaliplatin + Capecitabine	No grade III + toxicities attributable to bevacizumab: grade III-IV diarrhea: 27%,
	Resch et al. [151]	2011	Phase II	8	Rectal cancer	45 Gy/Capecitabine	Discontinued due to tox after 8 Pt., Grade III GI bleeding: 25%, Grade III diarrhea: 25%
	Kelly et al. [71]	2010	Case reports	3	Glioblastoma		Optic neuropathy
	Vargo et al. [152]	2011	Case report	1	Glioblastoma		Dural venous thrombosis

N-number of patients

Altogether, the combined use of bevacizumab and radiotherapy seems to be associated with a considerable risk of side effects (wound dehiscence, bleeding, fistula or GI complications). However, in selected cases the combination was feasible and even favourable concerning overall survival (retrospective) [69] and progression-free survival [72].

### Anti CD20 monoclonal antibody-rituximab

Rituximab is a monoclonal antibody directed against the CD20 antigen. It was initially developed and approved as a targeted agent for the treatment of CD20-positive non-Hodgkin lymphoma. In this setting, rituximab is mostly used in combination with chemotherapy (e. g. CHOP). Apart from the use of rituximab in oncology, its use has been extended to the treatment of refractory autoimmune diseases (e. g. rheumatoid arthritis, multiple sclerosis, systemic lupus erythematosus and idiopathic thrombocytopenic purpura among others).

The application of rituximab in combination with or shortly before/after radiotherapy of non-Hodgkin lymphoma has been prospectively studied [73-75]. So far no significant additional toxicities have been reported. All side effects seen in the trials have been attributed to the individual therapeutic modalities respectively [76]. Thus, at present the combination of rituximab with radiation does not seem to harbour any relevant risks.

#### Small molecules/tyrosine-kinase inhibitors (TKI's)

TKI's are small molecules able to pass the cell membrane and to inhibit intracellular tyrosine kinases of several growth factor receptors. Relevant examples are sunitinib, sorafenib, erlotinib or gefitinib. At present, TKI are used for diverse cancer entities and various clinical settings. Key indications are: Metastasized lung cancer/renal cell cancer/pancreatic cancer, locally advanced and metastatic breast cancer as well as hepatocellular carcinoma.

Up to now, no TKI has been approved for the simultaneous use with radiotherapy.

All toxicity data on combined toxicity are limited to case reports or studies with small numbers of patients. The clinical indications and the most common adverse effects of clinically used TKI's are summarized in Table 3.

**Table 3:** Studies on small molecules.

Substance	Author(s)	Year	Study type	N	tumour	RT dose/ChTx/technique	Toxicity
Sunitinib	Chi et al. [153]	2010	Phase II	23	HCC	52.5 (15 Fx) IMRT/3D-CT	9% grade III upper GI bleeding, 4% hepatitis grade III/4% grade III pancreatitis, 26% grade III + IV thrombopenia, 4% grade III leukopenia
	Stähler et al. [154]	2010	Prospective, non-randomized	22	RCC (all sites)	Median 40 Gy (median 8 Fx)	5% grade IV hypertension, 14% grade III + IV nausea
	Kao et al. [80]	2009	Phase I	21	Oligometastases	40-50 Gy (10 Fx)	62% grade III + IV lymphopenia, 19% grade III neutropenia, 14% grade III + IV thrombopenia, 5% grade III rectal bleeding
	Hui et al. [155]	2010	Phase II	13	Nasopharyngeal carcinoma	Sunitinib after RT (60-70 Gy) and multiple Chx	15% fatal hemorrhages, 31% Grade IV hemorrhages, (among other toxicities)
sorafenib	Peters et al. [77]	2008	Case study	1	RCC	8 Gy single dose	Grade V bowel perforation (stopped two days before, three days later recommenced)
lapatinib	Harrington et al. [156]	2009	Phase I	31	LA-HNSCC	66-70 Gy + CisPt	35% grade III mucositis, 19% grade III dermatitis, 13% grade III + IV lymphopenia, 6% grade III neutropenia
gefitinib	Cohen et al. [157]	2010	Phase I	69	LA-HNSCC	IC Carbo/Tax, hydroxyurea + 5-FU, 2 × 1.5 Gy/d	29% grade III + IV neutropenia, 86% grade III + IV mucositis, 33% grade III + IV dermatitis, 3% grade V infections, 17% grade III + IV infections, 4% grade III rash, 3% grade III neurotoxicity
	Pollack et al. [158]	2011	Phase II	43	Brainstem glioma in children	55,8 Gy	Grade III+IV: lymphopenia (21%), neutropenia (2%), GI (12%), infection (7%), n, pulmonary (5%), renal, skin (2%), metabolic (2%), intratumoral hemorrhage: 7%.
	Valentini et al. [159]	2008	Phase I/II	41	Rectal cancer	45 Gy + 5.4 Gy + 5-FU	20% grade III + IV GI toxicity, 15% grade III + IV skin toxicity, 39% grade III + IV hepatic toxicity, 10% grade III + IV GU toxicity, 7% other toxicities grade III + IV
	Wang et al. [160]	2011	Prospective	26	Stage III/IV NSCLC	Median 70 Gy	Grade IV leukopenia 4%, grade IV thrombopenia 8%, grade III esophagitis 4%, grade III pneumonitis 4%
	Zhang et al. [161]	2009	Phase I	24	NSCLC	54-60 Gy	4% grade III nausea
	Chen et al. [162]	2007	Phase I	23	HNSCC	Up to 72 Gy	13% grade III dermatitis, 57% grade III + IV mucositis, 39% grade III + IV dysphagia, 17% grade III + IV diarrhea, 30% grade III + IV neutropenia, 9% grade III + IV anemia, 4% grade IV tumour hemorrhage, 4% grade III GI bleeding
	Maurel et al. [163]	2006	Phase I	18	Pancreatic cancer	45 Gy	No DLT, 11% grade III + IV neutropenia, 6% anemia grade III
	Czito et al. [164]	2006	Phase I	16	Pancreatic + rectal cancer	50.4 Gy (1.8 Gy) + capecitabine	31% grade III + IV diarrhea
	Center et al. [165]	2010	Phase I	16	NSCLC	70 Gy 3D-CT + docetaxel	27% grade III + IV hematotoxicity, 27% grade III + IV esophagitis, 20% grade III + IV pulmonary toxicity
	Schwer et al. [166]	2008	Phase I	15	Malignant glioma	SRS 18-36 Gy	No DLT, no grade > II
	Olsen et al. [167]	2009	Phase I	12	Pancreatic cancer	50.4 Gy (1.8 Gy)	45% grade III nausea
erlotinib	Brown et al. [168]	2008	Phase I/II	79	GBM	60 Gy + TMZ	18% grade III/IV rash, 16% grade III/IV fatigue, 24% grade III/IV thrombopenia, 4% grade III nausea, 8% grade III diarrhea, 28% grade III/IV leukopenia, 3% grade III anorexia, 18% grade III/IV neutropenia, 6% grade III anemia, 14% grade III lymphopenia, 1% grade V infection without neutropenia, 5% grade III infection without neutropenia, 6% grade III/IV dyspnea, 1% grade III/IV keratitis, 1% grade V pneumonitis, 6% grade III/IV pneumonitis
	Prados et al. [169]	2009	Phase II	65	GBM	59.4 Gy/60 Gy + TMZ	43% grade III lymphopenia, 3% grade IV neutropenia, 2% grade IV thrombopenia, 8% grade III/IV fatigue, 2% grade III diarrhea, 6% grade III rash
	Herchenhorn et al. [170]	2010	Phase I/II	37	LA-HNSCC	70 Gy + Cis	No DLT
	Choong et al. [171]	2008	Phase I	34	NSCLC Stage III	66 Gy (2 Gy), Arm A: Erlotinib + Cis/Eto, Arm B: Induction Carbo/Tax, Carbo/Tax + Erlotinib	41% grade III + IV WBC, 32% grade III + IV neutropenia, 21% grade III + IV thrombopenia, 26% grade III + IV esophagitis, 3% grade III + IV vomiting, 6% grade III + IV diarrhea, 3% grade III/IV pneumonitis/ototoxicity
	Peereboom et al. [172]	2010	Phase II	27	GBM	60 Gy + TMZ	7% grade V febrile neutropenia, 4% grade V sepsis without neutropenia, 4% grade V PCP, a pt grade III neutropenia, 15% grade III neutropenia, 30% grade III + IV thrombopenia, 56% pts grade III lymphopenia, 15% grade III + IV anemia, 7% grade III fatigue
	Chang et al. [173]	2011	Retrospective	25	NSCLC	40-50 Gy	4% grade III rash, 4% grade III diarrhea/esophagitis/anemia, 8% grade III neutropenia/thrombopenia, 8% grade V pneumonitis, 4% grade III pneumonitis
	Li et al. [174]	2010	Phase II	24	LA esophageal cancer	60 Gy + Carbo/Tax	17% ≥ grade III leukopenia, 8% thrombopenia ≥ grade III
	Broniscer et al. [175]	2009	Phase I	23	GBM	54-59.4 Gy	39% grade III + IV lymphopenia, 4% grade III rash, 4% grade III diarrhea
	Robertson et al. [176]	2009	Phase I	22	Pancreatic cancer	Gem weekly, 30-38 Gy	5% grade III vomiting/fatigue/nausea, 5% grade III vomiting/diarrhea/nausea, 9% grade IV fatigue
	Duffy et al. [177]	2008	Phase I	20	Pancreatic cancer	50.4 Gy + Gem	100% grade III lymphopenia, 25% grade III thrombopenia, 30% grade III neutropenia, 5% grade IV neutropenia, 10% grade III anemia, 5% grade III fatigue, 15% grade III diarrhea, 10% grade III rash
	Krishnan et al. [178]	2006	Phase I	20	GBM	60 Gy	15% grade III stomatitis, 5% grade III fatigue/diarrhea
	Iannitti et al. [179]	2005	Phase I	17	LA Pancreatic cancer	50.4 Gy + Tax/Gem	6% grade III nausea/fatigue/rash/small bowel stricture/thrombopenia (each), 18% grade III dehydration/thrombosis, 12% grade III diarrhea/hypersensitivity, 6% grade IV neutropenia
	Nogueira-Rodrigues et al. [180]	2008	Phase I	15	LA cervical cancer	45 Gy + brachyTx + CisPt	7% grade IV hepatotoxicity, 7% grade III dermatitis, 20% III diarrhea, 13% grade III rash
	Arias de la Vega et al. [181]	2011	Phase I	13	LA-HNSCC	63 Gy + Cis adjuvant	Grade III/IV: Mucositis 54%, Asthenia 15%, skin 23%, diarrhea 15%.
	Lind et al. [182]	2009	Phase I	11	NSCLC, brain metastases	WBRT (30 Gy)	9% grade III rash/fatigue
	Dobelbower et al. [183]	2006	Phase I	11	Esophageal cancer	50.4 Gy + 5-FU	36% pts grade III + IV leukopenia, 9% grade III anemia, 9% grade III thrombopenia, 18% grade III + IV neutropenia, 27% grade III dehydration, 9% grade III nausea, 9% grade III/9% grade IV esophagitis
	Silvano et al. [82]	2008	Case report	1	NSCLC	2 × 8 Gy	Death caused by fatal diarrhea
	Huang et al. [184]	2008	Case study	1	NSCLC	WBRT 37.5 Gy	Death caused by exacerbated radiodermatitis and subdural hemorrhage
mTOR inhibitors (Sirolimus)	Sarkaria et al. [185]	2007	Phase I	7	NSCLC	60 Gy + CisPt weekly	14% grade III dysphagia, esophagitis, febrile neutropenia, pneumonia
	Bourgier et al. [186]	2011	Case reports	3	Breast/prostate/ovary cancer	45 Gy/70 Gy, later	Gastrointestinal radiation recall syndrome with everolimus/temsirolimus

N-number of patients, pt(s)-patient(s), n. r.-not reported, ChTx-chemotherapy, HCC-hepatocellular carcinoma, RCC-renal cell cancer, GBM-glioblastoma multiforme, DVT-deep vein thrombosis, Fx-fractions, SRS-stereotactic radiosurgery, DLT-dose limiting toxicity, LA-locally advanced, Gem-gemcitabine, Tax-Taxol (paclitaxel), Tx-therapy, TMZ-temozolomide, PCP-Pneumocystis pneumonia, Cis-cisplatin, Eto-etoposide,

When using sunitinib or sorafenib alone, mainly diarrhea, hypertension, fatigue, hand-foot syndrome, bleeding and hematotoxicity may occur as side effects. Concerning combined use with radiotherapy, one case report described a lethal small bowel perforation after 1x 8 Gy in a palliative setting, sorafenib had been stopped 2 days before and three days after radiotherapeutic treatment [77]. In another case, a lethal bronchial fistula occurred after radiation of the mediastinum [78]; as this phenomenon has been observed after sunitinib alone [79] no definite causality can be deduced. Furthermore, elevated bone-marrow toxicity was observed if large volumes of bones or liver were radiated; a phase I study concluded to avoid the combination with sunitinib when radiating volumes of more than 6 ccm of the liver. A dose reduction of sunitinib was advised for the following phase II study [80].

In patients with cerebral metastases increased intracerebral bleeding has been reported, this appears to happen with or without radiotherapy [81].

Concerning the simultaneous use of gefitinib/erlotinib and radiotherapy one case of fatal diarrhea after combining erlotinib with RT in the abdomen (2x8 Gy, q1w) has been reported [82]. And again, in patients with cerebral metastases increased intracerebral bleeding has been reported, however, this appears to happen with or without radiotherapy [83].

As long as no reliable data concerning the safety of the combination of TKI's and radiotherapy are available, such therapies should be used very carefully, especially if the above reported organs received relevant radiation doses. So far it is unclear if the increased intracerebral bleeding rates are induced by the combined treatment or by the drug alone. However, because of the severity of this adverse effect, special caution is warranted for combined treatment schedules. The same applies for tumours that tend to bleed outside of the brain. Radiotherapy in the abdomen or the pelvis together with TKI's might lead to an increased toxicity, including the occurrence of ulcera and bleeding.

#### mTOR inhibitors

According to pre-clinical data, an improvement of tumour growth by simultaneous administration of temsirolimus with radiotherapy seems possible [84-86]. However, the only study on long-term local tumor control revealed no beneficial effect regarding the combined treatment [84]. Preclinical data show an inhibition of vascular growth when combining everolimus with radiation, however a direct radio-sensitizing effect could not be consistently shown [85,86]. A recent study [87] showed evidence for a suppressed dsDNA break repair by everolimus.

Concerning toxicity, there is one phase I study using temsirolimus with topotecan in recurrent gynaecological malignancies [88]. Dose-limiting toxicity of this combination was myelo-suppression. Although this toxicity cannot be attributed to temsirolimus, we advise caution when combining mTOR-inhibitors with concomitant or sequential radiotherapy, especially if large volumes of bone are in-field as the latter is already known to potentially cause myelo-suppression.

Nevertheless there are no sufficient clinical data to adequately judge the risks and potential benefits of a combined use of mTOR-inhibitors with radiotherapy. As long as this is the case, it can be assumed that-similar to anti-angiogenic substances-the combinational use may lead to wound healing deficits, increased bleeding and thrombosis.

### Lenalidomide/thalidomide

Data on available studies combining radiotherapy and lenalidomide or thalidomide treatment are shown in Table 4. Thalidomide was initially used and approved as sedative drug until the early 1960s when it became clear that the intake of "Contergan" during pregnancy could lead to severe deformities. It was only in the late 1990s that thalidomide was rediscovered for its anti-angiogenic properties in cancer therapy [89]. Thalidomide is clinically used in the treatment of multiple myeloma; other areas of possible clinical use and ongoing clinical trials include leprosy, erythema nodosum leprosum and myelodysplastic syndrome.

**Table 4:** Studies on thalidomide and derivatives.

Substance	Author(s)	Year	Study type	N	tumour	RT dose/ChTx/technique	Toxicity
Thalidomide	Knisely et al. [93]	2008	Phase III	332 (90 thalidomide)	Brain metastases	37,5 Gy (2,5 Gy)	53% interruptions because of side-effects
	Chang et al. [187]	2004	Phase II	67	GBM	60 Gy (2 Gy), TMZ concomitant	10% grade III + IV neutropenia, 1% grade V; 16% grade III + IV thrombopenia, 9% grade III + IV rash, 1% grade III constipation, 9% grade III fatigue
	Atkins et al. [188]	2008	Phase II	39	CNS metastases (melanoma)	30 Gy (3 Gy) WBRT + TMZ concomitant	10% grade III + IV + V thrombosis, 5% grade III + IV + V myelosuppression, 8% grade III + IV + V cardiac events
	Ch'ang et al. [189]	2011	Phase II	24	HCC	50 Gy (2 Gy)	54% rash, 38% somnolence, 33% constipation
	Turner et al. [97]	2007	Phase II	13	Brainstem glioma + GBM	55.8 Gy (1.8 Gy)	8% grade IV DVT, ≥ 15% grade III leukopenia/motoneuropathy/constipation
Lenalidomide	Drappatz et al. [101]	2009	Pilot study	23	GBM	60 Gy (2 Gy)	4% grade IV pneumonitis/hypoxia, 9% grade III nausea, 4% grade IV pulmonary embolism, 4% grade III pneumonia

N-number of patients, pt(s)-patient(s), n. r.-not reported, ChTx-chemotherapy, LA-HNSCC-locally advanced head-and-neck cancer, GBM-glioblastoma multiforme, DVT-deep vein thrombosis

The most common side effects of thalidomide-besides somnolence-are thromboembolic events as well as peripheral polyneuropathy.

In vitro studies with cells of squamous cell carcinoma and of multiple myeloma showed no evidence for any radio-sensitizing quality of thalidomide. However, a radio-sensitizing effect has been observed in normal hematopoietic bone marrow [90]. Experiments in mice showed thalidomide induced tumour re-oxygenation pointing to a possible radio-sensitizing effect in vivo [91]. Experiments in rats indicate that thalidomide might be protective against radiation-induced proctitis when given 7 days after a single-RT [92].

In humans, thalidomide has been tested in combination with radiotherapy in phase I-III studies. Most data exist for radiation of the CNS combined with the administration of thalidomide.

The largest study so far was conducted by Knisely and co-workers [93]. In this phase III study 183 patients with multiple cerebral metastases were randomized for palliative WBRT (37,5 Gy in 15 fx) vs. WBRT (same dose and number of fractions) with thalidomide. In this study, only the known side effects of thalidomide occurred in the usual frequency. Hints to a possible interaction with radiotherapy have not been reported. Nearly half of the patients discontinued the study in the thalidomide arm due to side effects. The major limiting side effect was somnolence [93].

In malignant glioma, thalidomide was used in combination with radiotherapy or radiotherapy plus temozolomide in primary or recurrent settings [94]. Intratumoural bleeding and thromboembolic complications have been reported. However, the rate of complications was not higher than the reported rates for thalidomide alone [95-97].

Other studies, combining radiotherapy of soft tissue/bone metastasis as well as pelvic tumours with thalidomide simultaneously or sequentially revealed no evidence for increased risks of acute or late side effects [99].

However, a single study using radiotherapy (66 Gy in 33 fx) combined with vinorelbine and thalidomide in NSCLC stage III was abrogated after 10 patients due to side effects (thromboembolic, 1 bradycardia II°) [100]. As in this study only known side effects of thalidomide occurred, it still remains unclear whether radiation including the lung or the heart leads to increased side effects when combined with thalidomide.

Altogether the combination of thalidomide with simultaneous or sequential RT does not seem to be critical. Only in cases when large volumes of the heart or the lung are exposed, a certain level of cautiousness should be advised.

#### Lenalidomide

Lenalidomide is a derivative of thalidomide. Thus, the anti-angiogenic effect and the adverse effects are to a large extent similar to thalidomide. However, it largely lacks the sedative side effect, making it better tolerable for patients. Leukopenia and thrombocytopenia have also been reported.

In Europe and the US it is only approved in combination with dexamethasone for the treatment of multiple myeloma as 2nd line therapy. There is only very limited data regarding the combination of lenalidomide and radiotherapy. A single phase I trial [101] in glioblastoma used lenalidomide with RT (60 Gy, 30 fx). Thromboembolic events, pneumonitis and elevation of transaminases have been reported. The maximal tolerable dose was reported to be 15 mg/m^2^, corresponding to the respective dose for monotherapy. Being chemically similar to thalidomide and having a similar profile of side effects, one can indirectly assume a similar pattern of interaction with radiation.

#### Imatinib

Imatinib is a tyrosine-kinase inhibitor (TKI) of bcr-abl, PDGFR alpha/beta and c-kit. The first successful clinical application of imatinib was in chronic myeloid leukaemia as the bcr-abl-fusion gene plays a crucial role in this disease. As GIS-tumours display a high number of c-kit-mutations, they are currently also treated with imatinib. Imatinib alone is usually well tolerated. Known adverse effects are diarrhea, nausea, vomiting, erythema, edema or the increase of transaminases; leukopenia or thrombopenia usually occur only in leukemic diseases. Grade III-IV toxicity is reported in fewer than 10% of the patients.

Several in vitro experiments showed a putative radio-sensitizing effect of imatinib [102]. Additionally it has been shown, that the proliferation of fibroblasts can be slowed down in vitro by imatinib [103]. This leads to speculations about a potential protective effect of imatinib with regard to radiation-induced fibrosis. Three in vivo experiments support this hypothesis [104-106].

Regarding the clinical use of radiotherapy and imatinib only limited data is available. Imatinib has been used in recurrent glioma after radiotherapy (one 112-patient-trial with imatinib alone after radiotherapy and three 30-40-patient trials in combination with hydroxyurea). Unexpected adverse effects pointing to an increased toxic profile for the sequential use have not been reported [107-110]. In another trial, 27 patients have been treated with imatinib after radiotherapy in prostate cancer without unexpected side effects [111].

There is only one clinical phase I study regarding the simultaneous application of imatinib to radiotherapy (55.8 Gy in 21 fx) in children with brainstem-tumours. Retrospectively compared to a similar collective, subclinical bleeding seemed increased, but no other unexpected toxicities have been reported [112]. Additionally, there are two case reports for the combinational approach [113,114]. Again, in both cases no unexpected side effects have been reported.

Altogether, sequential application of imatinib with radiotherapy might not bear an increased risk for adverse effects. For the simultaneous application the limited amount of data does not allow a valid judgement about potentially increased side effects.

## Discussion

Radiotherapy combined with molecular targeted agents may be associated with unforeseen yet specific toxicities. Based on putative interactions of radiotherapy and the given agent with the targeted signalling cascade, any interactions may not only interfere with any anti-tumour efficacy but may also increase side effects. On the other hand, also radio-protective effects for the tumour are possible if new combined treatment schedules are used. Examples are cetuximab in multimodal radio-chemotherapy regimens for rectal cancer [115-119] or erythropoietin, which was thought to increase the haemoglobin level in head-and-neck cancer patients, but decreased survival most likely due to EPO-receptors on the cancer cells which were not known as a proliferative factor for tumours before [120,121].

However, there are still clinical situations where patients may benefit from the application of a targeted drug in combination with radiotherapy outside approved treatment schedules or clinical trials. The best example is a palliative systemic treatment for disseminated metastases and at the same time an indication for palliative or symptomatic radiotherapy of a single region. In this case, interruption of the systemic treatment may lead to systemic progression under radiotherapy. The present work aims to provide a helpful tool for clinical treatment decisions in such situations.

At present, only limited data is available on the interactions of targeted agents and radiotherapy. Data on toxicity are mostly derived from small case series, retrospective analyses or at best cohort and few randomized studies. For most substances, mild complications are reported-however, rarely exceptional fatal complications have been documented.

Overall, for any of the drugs mentioned here indications for a combination with radiotherapy have to be made cautiously (is a sequential treatment possible?). Furthermore, patients have to be questioned very specifically regarding the intake of targeted drugs. Frequently patients have been advised that these drugs are not "classical cytostatic drugs". Thus patients often do not self-report intake of targeted drugs when counselled for radiotherapy.

Simultaneous applications of targeted drugs during radiotherapy in non-established schedules should be an exception and reserved for those patients where the systemic tumour situation mandates rapid treatment. Whenever possible, large volume radiotherapy plus targeted drugs should be avoided. These remarks are especially important for hypo-fractionated regimens where high toxicities have been observed (in part with fatal consequences).

In conclusion, molecular targeted agents should only very cautiously in combination with radiotherapy. A meticulous and careful balancing of benefits and risks of increased toxicity is advised.

## Abbreviation

CNS: central nervous system; CT: chemotherapy; DFS: disease-free survival; EBRT: external beam radiotherapy; EGFRi: epidermal growth factor receptor inhibitor; IGRT: image-guided radiotherapy; KPS: Karnofsky Performance Status; mo: months; mPFS: median progression-free survival; MST: median survival time; MTD: maximum tolerated dose; mTOR: mammalian target of rapamycin.; mTTP: median time to progression; NTD: normalized total dose; OS: overall survival; PFS-6/-12: progression-free survival rate at 6/12 months; pt(s): patient(s); PTEN: phosphatase and tensin homolog deleted on Chromosome 10; QOL: quality of life; RCHT: radio-chemotherapy; RT: radiotherapy; VEGF-R: vascular endothelial growth factor receptor; WBRT: whole brain radiotherapy; wk: week.

## Competing interests

The authors declare that they have no competing interests.

## Authors' contributions

MN and CM performed the literature search and wrote the manuscript. MK, CMR and WB performed critical revision. CB participated in the conception as well as the preparation of the manuscript. All authors read and approved the final manuscript.
